# Nicotinic Receptor β2 Determines NK Cell-Dependent Metastasis in a Murine Model of Metastatic Lung Cancer

**DOI:** 10.1371/journal.pone.0057495

**Published:** 2013-02-28

**Authors:** Junwei Hao, Fu-Dong Shi, Mohammed Abdelwahab, Samuel X. Shi, Alain Simard, Paul Whiteaker, Ronald Lukas, Qinghua Zhou

**Affiliations:** 1 Tianjin Lung Cancer Institute, Tianjin Medical University General Hospital, Tianjin, China; 2 Department of Neurology, Tianjin Neurological Institute, Tianjin Medical University General Hospital, Tianjin, China; 3 Departments of Neurology, Neurobiology, Barrow Neurological Institute, St. Joseph’s Hospital and Medical Center, Phoenix, Arizona, United States of America; 4 Department of Chemistry and Biochemistry, Université de Moncton, Moncton, New Brunswick, Canada; Istituto Superiore di Sanità, Italy

## Abstract

Cigarette smoke exposure markedly compromises the ability of the immune system to protect against invading pathogens and tumorigenesis. Nicotine is a psychoactive component of tobacco products that acts as does the natural neurotransmitter, acetylcholine, on nicotinic receptors (nAChRs). Here we demonstrate that natural killer (NK) cells strongly express nAChR β2. Nicotine exposure impairs the ability of NK cells to kill target cells and release cytokines, a process that is largely abrogated by nAChR β2 deficiency. Further, nicotinic suppression of NF-κB-induced transcriptional activity in NK cells is dependent on nAChR β2. This nAChR subtype also plays a large role in the NK cell-mediated control of melanoma lung metastasis, in a murine lung metastasis model exposed to nicotine. Our findings suggest nAChR β2 as a prominent pathway for nicotine induced impairment of NK cell functions which contributes to the occurrence of smoking-related pathologies.

## Introduction

Smoking related disorders such as infection and tumorigenesis have been linked to the compromised functions of the immune system in smokers [1], [2]. Among the multiple immune-modifying components of tobacco smoke, nicotine has been shown to have a profound impact on a number of nicotinic acetylcholine receptor (nAChR)-bearing leukocytes from both innate and adaptive immune systems. Expression of nAChR α7 on macrophages and monocytes, and its ability to inhibit the immune response during systemic inflammation and in organ-specific diseases have been relatively well described [3], [4], [5], [6], [7], [8]. Results suggest that nicotine regulates the intensity of endotoxemia and sepsis [3], [4], [5], and attenuates -specific autoimmune responses in an nAChR α7-dependent manner [6], [7], [8]. On the other hand, it has recently been demonstrated that other nAChR subtypes may play a role in nicotine’s anti-inflammatory effects [3], [4], [5], [6], [7], [8]. In this context, the expression profile of additional nAChRs on leukocytes and their role in disease are relatively less explored.

NK cells are large, granular lymphocytes that operate through cytolytic activity and cytokine secretion. These two functions empower NK cells in innate host defense against certain microbial agents and cells undergoing malignant transformation. Several studies have shown that NK cell numbers and activities are decreased in smokers compared with non-smokers [1], [2]. Exposure to cigarette smoke attenuates the cytotoxic activity and cytokine production of NK cells in humans and mice [9], [10], [11], thereby linking NK cell defects to increased infection and cancer. Smoking has been particularly associated with the highly malignant small cell lung cancer. Even after surgical removal at an early stage, nearly half of patients die from a secondary tumor metastasis. It is postulated that this is due in part to defective NK cell-mediated immune surveillance because aberrant NK cell function in smokers increases the re-emergence of cervical cancer metastasis [12].

Here, we comprehensively examined the cellular and molecular effects of nicotine as one of the components of cigarette smoke on NK cells. We profiled nAChR expression on NK cells and identified nAChR β2 as a key determinant for nicotine-mediated impairment of NK cell functions. Further, we demonstrate that nicotinic inhibition of NK cell functions via nAChR β2 significantly increases melanoma metastasis in a xenogeneic model.

## Materials and Methods

### Animals

Female C57BL/6 mice (6–8 wk old), RAG2^–/–^, RAG2^–/–^γ_c_^–/–^, all on a C57BL/6 background, were purchased from Taconic Farms. α7 and β2 KO mice [13], also crossed to C57BL/6 background, were kindly provided by Dr Allan C. Collins. Mice were maintained under pathogen-free conditions. The Animal Research Ethics Board of Tianjin Medical University and St. Joseph’s Hospital and Medical Center approved all experiments described in this study.

### mRNA Purification and Reverse-transcription PCR

mRNA was purified from fresh acutely-isolated cells (∼1.5×10^6^ cells per sample) by using the µMACS mRNA isolation kit (Miltenyi Biotec), as per the supplied protocol. Reverse transcription was performed with the SuperScript III First Strand cDNA Synthesis kit (Invitrogen, USA) by following the supplied protocol. Oligo-dT sequences were used to prime the reverse transcriptase. PCR was then performed following established protocols, using a variety of primers that are specific to each target mRNA (Table 1). Primer pairs were designed with the use of PubMed’s “Primer-blast” tool (http://www.ncbi.nlm.nih.gov/tools/primer-blast/). For each pair, forward and reverse primers were specific to different exons, so that potential DNA contamination could be ruled out. PCR was performed using the RedTaq PCR kit (Sigma, USA), according to the supplied protocol. Primers were always tested with total brain cDNA (positive control), and the optimal annealing temperature (Tm) was determined empirically (with the use of a Temperature gradient thermocycler), while maintaining high stringency conditions. A negative control (no cDNA template), was included in every set of reactions. PCR products for each primer pair from positive controls were sequenced in order to ensure the accuracy of our results.

**Table 1:** Primers used to detect nAChR subunits.

nAChR	NCBI	Forward Primer	Reverse Primer	Product	Tm
Subunit	Template	Sequence	Sequence	Size (bp)	(°C)
α3	NM_145129.2	gccaacctcacaagaagctc	atgtggggtttagcagcaac	680	58
α4	NM_015730.5	cagtagccaatatctcagat	gtagaacagtggcagtcgg	577	55
α5	NM_176844.3	gggttcgtcctgtggaacacctga	ggtcctgtaggattatatcg	431	58
α6	NM_021369.2	tgttccagcagataacatctg	tgaattgaacactctcgatg	987	55
α7	NM_007390.3	cgtgggcctctcagtggtcg	ggccatgaggcacaagcggt	514	58
α9	NM_001081104.1	ctatttccccttcgacag	ttttgtcagtgcttcatagc	798	55
β2	NM_009602.3	ctccaactctatggcgctgct	cgtcggcctggcagtgcgat	623	58
β3	NM_173212.3	acggagagtaagggaaccgt	accagcagccctcagttcta	364	58
β4	NM_148944.4	tctctgttcgctctgcttca	acacagtggtgacgatggaa	913	58

Primer pairs were designed with the use of PubMed’s “Primer-blast” tool (http://www.ncbi.nlm.nih.gov/tools/primer-blast/). For each pair, forward and reverse primers were specific to different exons, so that potential DNA contamination could be ruled out. The optimal annealing temperature (Tm) was determined empirically with the use of a temperature gradient thermocycler, while maintaining high stringency conditions. PCR products for each primer pair were sequenced in order to ensure the accuracy of our results.

### Overall nAChR Expression Assessed by Radioligand Saturation Binding

Epibatidine, which is selective for β2- and β4-containing nAChRs (which can assemble with α4, α5, α6 and β3 subunits), was used to assess the amount of β2-containing nAChRs, as done previously [14], [15]. In order to maximize assay sensitivity, we used high specific activity [^125^I]epibatidine (I-Epi; 2200 Ci mmol^−1^; PE Life Sciences, Waltham, MA). To this end, purified NK cells were used immediately after isolation. Cells were resuspended in isotonic binding buffer (mM: NaCl, 144; KCl, 2; CaCl_2_, 2; MgSO_4_, 1; HEPES 20; pH = 7.5), supplemented with bovine serum albumin (0.1% (w/v)). Incubations were performed at 22 °C for 2 h in 96-well plates. Samples were incubated with a range of eight concentrations (6.25 – 800 pM) of I-Epi in a total volume of 30 µl of supplemented isotonic binding buffer. Total (no peptide added) and non-specific binding (defined using 100 mM carbamycholine) were measured at each concentration, in triplicate. To differentiate β2- from β4-containing nAChRs, 10 nM A85380 was added in conjuncton with 200 pM I-Epi [14]. Binding reactions were terminated and washed by filtration using a 96-place manifold. Particulate fractions were collected onto single layers of Inotech 0.75 µm retention glass fiber filters soaked in 0.5 % polyethyleneimine. Bound radioligand were quantified using a Microbeta plate counter (PerkinElmer Life Sciences, Wellesley, MA).

### Nicotine Treatment in vivo and in vitro

For in vivo delivery, a 100 mg/ml solution containing (-)nicotine bitartrate (Sigma, St. Louis, MO, USA) in phosphate-buffered saline (PBS) or a solution of PBS alone was freshly prepared 24 h before pump implantation and loaded into Alzet® osmotic minipumps (model 2006, Durect Corporation, Cupertino, CA, USA). The pumps were implanted subcutaneously on the right side of the back of the mouse and continuously delivered either PBS or nicotine salt at 3.6 µl/d for 6 weeks, and then the pumps were removed. This equated to delivery of 0.39 mg of nicotine free base per mouse per day. For an ∼30 gm mouse, which is at the upper end of weight for animals used in the study, this equates to ∼13 mg of nicotine free base/kg/d or ∼0.54 mg of nicotine free base/kg/hr. Plasma nicotine levels in mice are ∼100–200 ng/ml (∼0.6–1.2 mM) after infusion of ∼2–4 mg/kg/hr of drug and ∼45 ng/ml (∼280 nM) after infusion at ∼0.5 mg/kg/hr. For comparison, human smokers have peak plasma nicotine levels of 10–50 ng/ml (∼60–310 nM). Thus, nicotine levels in plasma (extrapolated to be ∼49 ng/ml or ∼300 nM) of mice used in the studies are comparable to those in the plasma of human smokers. Some control mice received PBS via direct injections rather than through minipumps, but either delivery method produced similar results. For the in vitro experiments, 0.1–100 µM/ml of nicotine was added to the cell cultures.

### B16 Melanoma Cell Line and Tumor Challenge

B16-F10-luc2 (B16) is a luciferase-expressing cell line from mouse melanoma (Caliper Life Sciences, Alameda, CA, USA). Mice of the indicated genotypes were challenged i.v. with B16 cells in 0.2 ml of PBS via the tail vein. Cell numbers used and times of sacrifice are referred to previously established protocols [9], [15], [16] and specified in the results section. Animals were anesthetized and euthanized via exsanguinations through abdominal vein puncture. Lungs were removed and placed in PBS. Tumor nodules were counted using a dissecting microscope by a researcher blinded to the treatment groups. In some cases, lungs were immersed in a 67% ethanol, 9% formaldehyde, and 4% glacial acetic acid solution and the number of tumor foci was enumerated 24–48 h later.

### Isolation of Lung Mononuclear Cells

Lung mononuclear cells were isolated as described previously [17]. Briefly, lungs were perfused with prewarmed HBSS from the right ventricle. Cell suspensions from excised organs were generated by collagenase digestion and followed by mechanical mincing. Cell debris were removed by passing through nylon mesh. Cells were washed and resuspended in HBSS. Nonparenchymal cells were isolated by density-gradient centrifugation with Lympholyte-M (Cedarlane Laboratories).

### NK Cell Cytotoxicity Assays

NK cell cytotoxicity was assessed by the chromium release assay using ^51^Cr-labeled YAC-1 murine lymphoma cell line [18] or B16 tumor cells. Freshly purified NK cells were incubated with ^51^Cr-labeled target cells (5 × 10^3^) at various effector cell/target cell ratios. After 4 h of incubation, the supernatants were harvested and ^51^Cr-release was measured with a γ-counter (PerkinElmer, Waltham, MA). The percentage of specific lysis was calculated according to the following formula: (experimental release – spontaneous release) / (maximum release - spontaneous release) × 100. Cytotoxicity assays were done in triplicate [18].

### NK Cell Proliferation Assays

NK cells were isolated from the spleen of mice that were treated with nicotine or PBS. Cells were suspended in culture medium containing Dulbecco’s modification of Eagle’s medium (Gibco, Paisley, UK) supplemented with 1% (v/v) minimum essential medium (Gibco), 2 mM glutamine (Flow Laboratory, Irvine, CA, USA), 50 IU/ml penicillin, 50 mg/ml streptomycin, and with 10% (v/v) FCS (all from Gibco). 4×10^5^ cells in 200 µl of culture medium were placed in each well of 96-well, round-bottom microtiter plates (Nunc, Copenhagen, Denmark). For the in vitro nicotine exposure experiments, nicotine (0.1–100 µM) was added to the culture medium in addition to LPS (10 µg/ml). After 3 days of incubation, the cells were pulsed for 18 h with 10 µl aliquots containing 1 µCi of ^3^H-methylthymidine (specific activity of 42 Ci/mmol; MP Biomedicals, Irvine, CA, USA) per well. Cells were harvested onto glass fiber filters and thymidine incorporation proportional to the degree of cell proliferation was then measured. The results are expressed as counts per minute (cpm).

### FACS Analysis

Single cell suspensions (10^6^ cells) were prepared from spleen or lymph nodes, and stained with fluorochrome-conjugated antibodies. All antibodies were purchased from BD Biosciences or eBioscience unless otherwise indicated. Antibodies were directly labeled with one of the following fluorescent tags: FITC, PE, PerCP, allophycocyanin (APC), PC5, or PC7. The following anti-mouse antibodies were employed: CD3 (145-2C11), NKG2A (20d5), NKG2D (CX5), Ly49 I (YLI-90), NK1.1 (PK136), perforin (eBioOMAK-D) and granzyme B (16G6). Flow cytometric data were collected on a FACSAria^TM^ flow cytometer (Becton Dickinson, Mountain View, MD) and analyzed with Diva^TM^ software. Isotype-matched negative control mAbs were used for all stains. To determine the percentage of cells producing selected cytokines, values obtained with isotype controls were subtracted from those with specific mAb.

### Tumor in vivo Imaging

Bioluminescence images were obtained using an IVIS Imaging System 200 Series (Caliper Life Sciences, Hopkinton, MA). For *in vitro* imaging, bioluminescent cells were serially diluted from 10^5^ cells in complete media into black, clear bottomed, 24-well plates (Costar, Acton, MA, USA). D-luciferin (Caliper Life Sciences, Hopkinton, MA.) at 150 µg/ml in media was added to each well 5–10 min before imaging. Imaging time was 1 min/plate. For *in vivo* imaging, Mice received an intraperitoneal injection of D-Luciferin 150-mg/kg (Caliper Life Sciences, Hopkinton, MA). Mice were then placed onto the warmed stage inside the light-tight camera box with continuous exposure to 1–2% isoflurane. Imaging times ranged from 1 s to 3 mints. Regions of interest from displayed images were identified around the tumor sites and were quantified as total photon counts or photons/s using Living Image® software (Caliper Life Sciences, Hopkinton, MA).

Tumor challenge experiments were conducted with melanoma B16 cells expressing luciferase. For quantitating tumor burden, at 10 minutes after the intraperitoneal injection of 3 mg/mouse D-luciferin (Caliper Life Sciences, Hopkinton, MA), mice were anesthetized and placed into the light tight chamber of an IVIS 200 bioluminescence imaging system (Caliper Life Sciences, Hopkinton, MA). Data was collected as photons/sec/cm^2^ using the Living Image® software (Caliper Life Sciences, Hopkinton, MA).

MRI was performed using a 7T small animal, 30-cm horizontal-bore magnet and BioSpec Avance III spectrometer (Bruker, Billerica, MA) with a 116 mm high power gradient set (600 mT/m) and a 72 mm whole-body mouse transmit/surface receive coil configuration. Axial and coronal T1-weighted (MSME; TE 10.5 ms, TR 322 ms, 0.5 mm slice thickness, matrix 256×256, field of view (FOV) 2.8 cm, eight averages, 40 coronal slices, scan time 22 minutes, and 20 axial slices, scan time 16 min) and fat-suppressed turbo spin echo T2-weighted (RARE; TE1 14.5 ms, TE2 65.5 ms, TR 4500 ms, 0.5 mm slice thickness, Matrix 256×256, FOV 2.8 cm, eight averages, 40 coronal slices, scan time 28 minutes, and 20 axial slices, scan time 28 minutes) images were acquired, covering the volume of brain from the olfactory bulb/frontal lobe fissure to the cervical spinal cord. MRI data were analyzed using the MEDx3.4.3 software package (Medical Numerics, Virginia, USA) on a LINUX workstation.

### Cytokine Quantification

Standard ELISAs for assessment of cytokine release by NK cells (IFN-γ, MIP1α, and GM-CSF) were performed using BD OptEIA ELISA kit (BD Biosciences Pharmingen, San Diego, CA) as previously described [18]. Results were expressed as mean cytokine concentration (pg/ml) ± SEM.

For intracellular cytokine staining, single cell suspensions were prepared and incubated at 37°C for four days in round-bottomed plates (2×10^6^ cells/well), and stimulated with a mixture of PMA (20 ng/ml), ionomycin (1 µg/ml), and brefeldin A (5 µg/ml) for another 5 h at 37°C. After harvesting, cells were stained for surface markers, fixed and permeabilized with Cytofix/Cytoperm kit (BD Biosciences), then stained with anti-IFN-γ mAb (XMG1.2) conjugated with Alexa 647. All samples were analyzed on a FACSAria^TM^ using Diva^TM^ software.

### qRT-PCR

Total RNA was extracted from cell suspensions of spinal cords using TRIzol (Invitrogen). First-strand cDNA of each sample was synthesized using a reverse transcription kit (Invitrogen). RT-PCR was performed as previously described, using an ABI Prism 7900-HT sequence system (Applied Biosystems) with the QuantiTect SYBR Green PCR kit (QIAGEN), in accordance with the manufacturer’s instructions. The following primers were used: NF-kB forward, 5′-ATTTGAAACACTGGAAGCACGG-3′; NF-kB reverse, 5′- CCGCCTTCTGCTTGTAGATAGG -3′; IKKα forward, 5′-TGCACACCGTGCAGAGTCA -3′; IKKα reverse, 5′- TGCTTGCAGCCCAACAACT -3′; hypoxanthine-guanine phosphoribosyltransferase (HPRT) forward, 5′-AGCCTAAGATGAGCGCAAGT-3′; and HPRT reverse, 5′-TTACTAGGCAGATGGCCACA-3′. The HPRT gene was amplified and served as an endogenous control. 1 µl of first-strand cDNA product was amplified with platinum Taq polymerase (Invitrogen) and gene-specific primer pairs. Each sample was assayed in triplicate and experiments were repeated twice. The relative amounts of mRNA were calculated by plotting the Ct (cycle number), and mean relative expression was determined by the 2^−ΔΔCt^ comparative method.

## Results

### nAChR Expression Profile on NK Cells and Confirmation by Ligand Binding Assays

**Figure 1:**
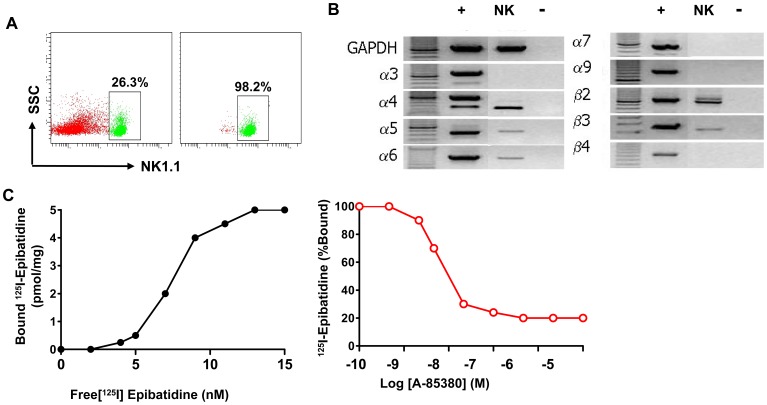
Nicotinic acetylcholine receptor (nAChR) expression on NK cells. Spleen, or lymph node cell suspensions were obtained RAG2^−/−^ mice, and FACS was performed as described in the *Materials and Methods* section. **A**. Representative dot plots of cell populations before and after sorting are shown in the left- and right-hand panels, respectively. The purity of NK cells reached >98% purity after sorting. **B**. mRNA was purified from the sorted NK cells, and cDNA was synthesized by reverse transcription. PCR was then performed using primers specific for each mouse nAChR subunit. A positive control (+), using whole mouse brain cDNA, and a negative control (-), without cDNA, were included in each reaction. For each nAChR subunit, bands from the positive control and at least one sample from each cell type were sequenced and confirmed as the correct PCR product. **C**. nAChR expression assessed by radioligand saturation binding. Data depicted are representative from 2 to 3 separate experiments with similar results.

To determine nAChR mRNA expression on NK cells, we sorted NK cells from pooled splenocytes of RAG2^−/−^ C57BL/6 mice that are devoid of T cells, NKT cells and B cells. mRNA was extracted from highly purified NK cells (Figure 1A) and nAChR expression profile was determined by PCR. Our results indicate that NK cells express nAChR α4, α5, α6, β2 and β3 but not α3, α7, α9; nor β4. Furthermore expression of β2 was particularly strong (Figure 1B). We then investigated whether nAChR proteins capable of engaging in nicotinic radioligand binding are assembled in NK cells by using an ^125^I-labeled epibatidine (I-Epi) binding assay (200 pM I-Epi +/− 100 mM carbamylcholine), as described elsewhere [14], [19], [20], [21]. NK cells (∼1 million) were purified by FACS, and mouse brain cortex homogenate was used as a positive control. Specific I-Epi binding was 99 fmol/mg protein in NK cells, about equal to specific binding levels in mouse whole brain (Figure 1C). These data demonstrate that NK cells express significant numbers of assembled nAChRs (as well as nAChR subunit mRNAs as shown previously) at levels that can be quantified with this assay. Since I-Epi binds to both β2- and β4-containing nAChRs [14], competitive binding with the nAChR β2-selective compound A85380 was used to discriminate between these two receptor subtypes. We found that A85380 largely abrogated I-Epi binding, demonstrating that the vast majority of nAChRs present on NK cells contain the β2 subunit (Figure 1C). Collectively, our results reveal that NK cells express an array of nAChR mRNAs and that most of these subunits assemble into β2-containing nAChRs. The strong expression of nAChR β2 in NK cells, both at the mRNA and protein levels suggest that β2-containing nAChRs are candidates for mediation of nicotine’s effects on NK cell activity.

### Influence of nAChR β2 Deficiency on Expression of NK Cell Receptors and Effector Molecules

**Figure 2:**
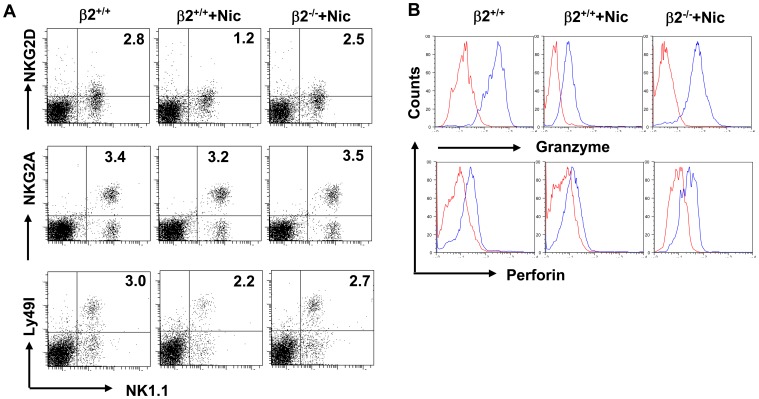
Nicotine alters NK cell phenotype via nAChR β2. Wild type (β2^+/+^) or nAChR β2 knock-out (β2^−/−^) mice received nicotine (Nic) or PBS for 21 days before they were scarified. Single cell suspensions were prepared from spleens. Frequency and phenotypes of NK cells within the mononuclear cells were analyzed by FACS. **A.** Expression of NKG2D, NKG2A and Ly49I on gated NK1.1^+^CD3^−^ cells. **B**. Expression of perforin and granzyme B secreted by gated NK1.1^+^CD3^−^ cells. The plot data represents results from two separate experiments (n = 3–4 mice/group). P values, Student’s *t*-test, *p<0.05.

NK cell functions are dictated by stimulatory and inhibitory receptors on NK cells. Activating signals are generated through NK group 2 member (NKG2D), which recognize the stress-inducible molecules MHC class I chain-related proteins (MICA and MICB) and the UL-16-binding proteins 1-4 (ULBP1-4), also known as RAET proteins, or through natural cytotoxicity receptors (NCRs) that recognize viral hemagglutinin and as yet undefined tumor-associated ligand. The inhibitory signals are generated by binding of MHC class I molecules to killer cell immunoglobulin-like receptors (KIRs) in humans, or to Ly49 in mice, in addition to the CD94/NKG2A heterodimer in both species [22], [23]. We compared the expression of several of these receptors on spleen NK cells isolated from nicotine- or PBS- exposed mice and investigated the influence, if any, of nAChR β2 deficiency. Expression of inhibitory receptor NKG2A is not significantly altered on NK cells by nicotine; whereas expression of NKG2D and Ly49I were reduced in mice that received nicotine, and such reductions are largely abrogated in nAChR β2^−/−^ mice (Figure 2A) [13]. Similar findings were obtained regarding expression of NK cell effector molecular Granzyme B and perforin (Figure 2B). We also observed that nicotine has a similar effect on lung-derived NK cells receptor profile and effector molecules (data not shown).

### nAChR β2 Deficiency Reverses the Inhibitory Effects of Nicotine on NK Cell Functions

**Figure 3:**
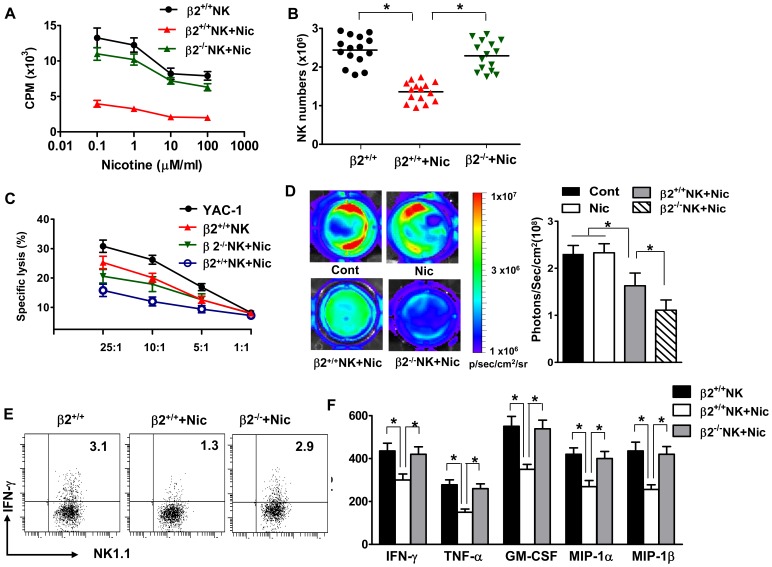
Nicotine impairs NK cell proliferation and killing of target cells by acting on nAChR β2. NK cells were sorted from splenocyte single cell suspensions of wild type (β2^+/+^) or nAChR β2 knock-out (β2^−/−^). **A**. Cells were then cultured in the presence of various concentrations of nicotine (0.1, 1, 10 or 100 µM), and [^3^H]thymidine incorporation was measured (10^3^ cpm + SEM; ordinate). **B**. NK cell numbers were assessed from β2^+/+^ or β2^−/−^ mice in the presence or absence of nicotine. (N = 6–8 per group; Student’s *t*-test, * p<0.05). **C**. ^51^Cr-labbed target cell YAC-1 were incubated with NK cells derived from β2^+/+^ or β2^−/−^ mice in the presence or absence of nicotine, at the indicated effector/target cell ratio. Killing of target cells are measured by ^51^Cr release assay. **D**. B16 cells were incubated with NK cells derived from β2^+/+^ or β2^−/−^ mice in the presence or absence of nicotine. D-Luciferin was added to each well and the plate was imaged to obtain photons/s per cell. Wells with cells alone (no nicotine) or cells (added nicotine) were included as controls. The killing of B16 cells are measured via bioluminescence imaging. The data of one experiment out of two performed is shown, n = 3–4 mice/group. P values, Student’s *t*-test, *p<0.05. **E**. Production of IFN-γ was measured by intracellular cytokine staining. Dot plots are representative of two separate experiments (n = 6–18 mice). **F**. Production of IFN-γ, TNF-α, GM-CSF, MIP-1α and MIP-β by sorted NK cells was measured by ELISA. P values, Student’s *t*-test, *p<0.05.

To understand the biological consequences of altered NK cell receptor expression induced by nicotine exposure, we investigated the effects of nicotine on NK cell mediated killing of target cells, as well as release of cytokines by NK cells. NK cells normal represent 5–10% of peripheral blood mononuclear cells in human and 1–3% of monocytes in mouse spleen or lymph node. For NK cells to perform their functions, these cells must proliferate under pathological situations. Thus, we first assessed the influence of nicotine on NK cell proliferation and found that nicotine reduced NK cell proliferation and numbers (Figure 3A, B). nAChR β2 deficiency largely restored the capacity of NK cells to proliferate and numbers in the presence of nicotine (Figure 3A, B).

Next, we assessed the influence of nicotine on NK cell lytic activity and potential roles for nAChR β2. Compared with control mice that received PBS, NK cells from nicotine-treated mice exhibited reduced cytotoxicity against YAC-1, a classic NK target cell with lower MHC class I expression (Figure 3C). Primary human and mouse melanoma, or cell lines, shared expression of ligands for natural cytotoxicity receptors (NCRs) and DNAX accessory molecule-1 (DNAM-1), the two emerging NK cell receptors key for cancer recognition [15]. Further, these cells have low MHC class I expression [15]. NK cells are thus able to recognize and lyse human and mouse melanoma cells in vitro and in vivo, and to prevent melanoma metastasis in adoptive transfer experiments [24]. We therefore used the murine B16 cell line, derived from spontaneous murine melanoma, and demonstrated that NK cells readily kill B16 tumor cells, and that this effect was significantly reduced when exposed to nicotine (Figure 3C, D). To investigate the contributions of nAChR β2 receptor to mediate the effects of nicotine, we perform these experiments using nAChR β2^−/−^ NK cells (Figure 3C, D). In all experiments, nAChR β2 deficiency significantly abrogated the effects of nicotine.

Last, we examine the impact of nicotine on cytokine production by NK cells. Compared with control mice, IFN-γ, GM-CSF, MIP-1α, MIP-1β and TNF-α secretion were reduced in mice received nicotine (Figure 3E, F). Again, these parameters were reversed to a great extent in nAChR β2^−/−^ mice . Collectively, our results demonstrate that nicotine impairs NK cell functions including proliferation, cytotoxicity toward YAC-1 cell and B16 tumor cells, and production of cytokines. Our findings also show that despite expression of multiple nAChRs, nAChR β2 emerged as a key mediator for nicotinic effects on NK cells, as deficiency of nAChR α7 fails to significantly alter the influence of nicotine on NK cells (data not shown).

### Nicotine Inhibits NF-κB Transactivation in NK Cells through nAChR β2

**Figure 4:**
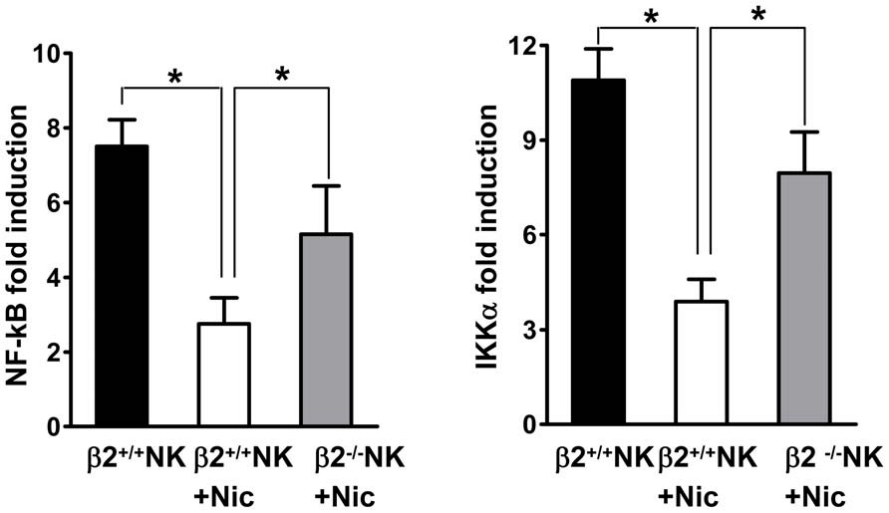
Nicotine inhibits NF-κB activation in NK cells through nAChR β2. NK cells were sorted from splenocyte single cell suspensions of RAG2^−/−^ and RAG2^−/−^β2^−/−^ mice. NF-κB and IKKα mRNA of sorted NK cells in β2^+/+^ or β2^−/−^ mice received nicotine (Nic) was measured by qRT-PCR. The data of one experiment out of two performed is shown, n = 3–4 mice/group. P values, Student’s *t*-test, *p<0.05.

Mobilization of the transcription factor NF-κB is an essential step for NK cell activation and proceeds its cytotoxicic activity and production of cytokines. To evaluate the impact of nicotine on NF-κB activity in NK cells, we sorted NK cells from the spleen of RAG2^−/−^ mice, and cultured the isolated NK cells alone or with nicotine for 24 h. We performed a series of real-time PCR experiments looking at changes in transcript abundance, and saw that addition of nicotine suppressed gene transcription of NF-κB, and IKKα, an alternative regulator of NF-κB in NK cells (Figure 4). To determine if nAChR β2 is involved, we cross-bred RAG2^−/−^ with nAChR β2^−/−^ mice to generate double knockout mice. When NK cells derived from these mice were used, the effect of nicotine on NF-κB and IKKα transcription becomes minimum(Figure 4). Thus, nAChR β2 mediates the effects of nicotine on NK cell transcription genes that are critical for their functions.

### Nicotine Exposure Promotes Lung Metastasis, a Process Dependent on nAChR β2

**Figure 5:**
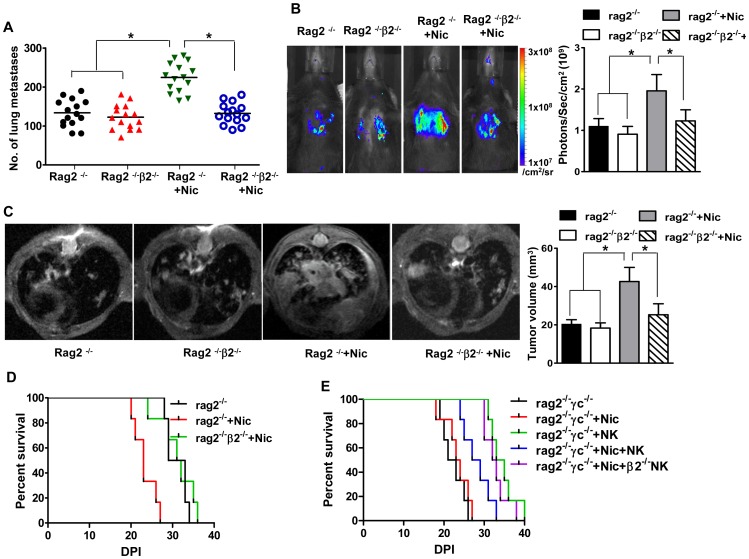
Impairment of NK cell-dependent suppression of B16 tumor cell metastasis by nicotine is mediated by nAChR β2. Mice of different genotypes received nicotine (Nic) or PBS for 21 days and engrafted with the B16 melanoma cell line (1×10^6^ cells/mouse). **A**. Seven or 14 days later, a portion of mice were euthanized and the lung dissected. Total numbers of melanoma nodules counted in these organs are shown (n = 12 mice/group). **B, C**. Quantification of tumor growth in these animals during the period are achieved via bioluminescence imaging (B) and high field MRI (C for T1 images; Figure S2 denotes Th2 images. N = 15–18/group. P values, ANOVA, *p<0.05. **D**. Survival for the remaining animals are monitored for 60 days (n = 15–18 mice/group). **E**. Mice were engrafted with melanoma cells lines (1×10^6^/mouse) in the presence or absence of nicotine, and NK cells (5×10^5^ NK cells/mouse) from wild type or nAChR β2^−/−^ mice. Mice were then monitored for survival up to 60 days. Results are pooled from three experiments with similar results. N = 6–8 mice/group.

Cigarette smoke has been experimentally demonstrated to increase lung metastatic tumor burden [11]. As multiple chemicals contained in cigarette smoke can have this effect, we sought to specifically address the role of nicotine exposure in this process. To this end, we adopted the murine B16 cell line derived from spontaneous murine melanoma. Because B16 tumor cells have high lung metastasis potential and such metastasis can be inhibited by NK cells, this model is ideal to address the role of nicotine and its receptors in this pathological process. For this purpose, C57BL/6 RAG2^−/−^ mice were treated with nicotine or PBS via osmotic pump implantation for 14 days and beyond. The dosage selection is based on smokers and justified in detail in the methods section and in our previous publications [7], [8]. These mice were then challenged i.v. with 1×10^6^ B16- melanoma cells. Based on the literature [11], [15], [16], we did preparation experiments in which tumor burden and survival rates were compared in mice receiving 1×10^5^, 2.5×10^5^, 5×10^5^, 1×10^6^ and 2.5×10^6^ B16 cells. We found that transplantation of 1×10^6^ provided the most efficient comparison of tumor burden, and thus this concentration was used for all subsequent experiments (Table S1). 14 days of nicotine or PBS exposure at the onset of tumor challenge lead to no significant difference in tumor burden between the two groups (data not shown). Nicotine was thus continuously released in these mice until 21 days post tumor challenge. Compared with control mice that received PBS, mice treated with nicotine had significantly greater lung tumor burden (Figure S1; Figure 5A), increased bioluminescence imaging (Figure 5B),tumor volume when assessed by high field MRI of both T1 (Figure 5C) and T2 imaging (Figure S2), as well as decreased survival rates (Figure 5D).

To determine the contributions of nicotinic receptors, we cross-breed RAG2^−/−^ with mice lacking nAChR α7 or β2, two nAChRs prominently expressed by NK cells, and performed similar experiments as described above. Lack of α7 receptors exerted little influence on B16 cell lung metastasis promoted by nicotine (data not shown). In contrast, promotion of metastasis by nicotine is largely diminished in RAG2^−/−^β2^−/−^ mice, as the tumor burden was indistinguishable between RAG2^−/−^ and RAG2^−/−^β2^−/−^ mice (Figure 5A–C). Thus, β2 receptor plays a critical role in mediating the effects of nicotine’s facilitation of lung tumor metastasis.

### nAChR β2 Determines NK Cell-dependent Tumor Metastasis

The effects of nicotine in promoting B16 cell line lung metastasis can be achieved via a direct effect on tumor cells, lung microenvironment, or immune cells. We focused our investigations on NK cells because of their known effects in constraining B16 cell metastasis, as well as strong expression of several nAChRs in these cells. In addition, as RAG2^−/−^ mice have no intrinsic T and B cells, the role for nAChR β2 in determining the effect of nicotine in tumor metastasis is likely via NK cells. We therefore first compared tumor burden and survival rates in RAG2^−/−^ and RAG2γc^−/−^ mice. Clearly, the lungs of RAG2^−/−^γc^−/−^ mice had larger tumor burden and majority of these mice quickly succumb to death after B16 cell challenge (Figure S1; Figure 5E). Our results thus confirm the role of endogenous NK cells in restricting B16 lung metastasis.

Next, we determined the role of nAChR β2 on NK cells in curbing tumor metastasis. For this purpose, we co-transfered B16 tumor cells with NK cells isolated from wild type and nAChR β2^−/−^ mice, into the recipient RAG2^−/−^γc^−/−^ mice. Lung tumor number and survival rates were indistinguishable between B16-recipient RAG2^−/−^γc^−/−^ treated with or without nicotine (Figure 5E). Thus significant roles for macrophage, dendritic cells and other cells in B16 lung metastasis are largely ruled out. Further, co-transfer of NK cells isolated from PBS-treated control mice improve the survival rate of recipient mice. However, such improvement is reduced markedly when NK cells derived from nicotine-treated mice are used for transfer experiments. Importantely, when transfer of NK cells isolated from β2^−/−^ mice, irrespective nicotine treatment, improved the survival rate (Figure 5E), whereas nicotine maintains its impact on B16 tumor cell metastasis when α7^−/−^ NK cells are co-transferred (data not shown) Therefore, our results suggest that nAChR β2 determines NK cell-dependent tumor metastasis.

## Discussion

Despite ongoing efforts to reduce smoking prevalence, over 1.1 billion people continue to smoke, and smokers in countries like China comprise a large portion of the overall smoking population, which is estimated about one-sixth of the world’s population [2]. Due to the prominent association of smoking with lung tumor and lung metastasis, we addressed several critical issues regarding cigarette smoke, immunity and tumor metastasis in a well established murine lung metastasis model following B16 melanoma challenge. The combined effects of enormous carcinogens, as well as multiple immune suppressive chemicals contained in cigarette smoke contribute to tumorigenesis. Until now, how nicotine, the psychoactive component of tobacco products, affects NK cells and their ability to perform immune surveillance against lung tumors, and which nAChRs mediate such events, were not known.

Several findings revealed by the current study are significant. First, we defined the influence of nicotine on the phenotype and functions of NK cells. Overall, we found that nicotine exposure reduces expression of NK cell-related activating signals as well as NK cell-associated immune effector molecules. This was associated with reduced NK cell proliferation, production of cytokines, cytolytic activity against target cells, and transactivation of NF-κB. Second, we are able to identify that among several nAChRs, the β2 subunit emerged as a principle mediator for these inhibitory effects of nicotine on NK cells. Third, we demonstrated the impact of nicotine on NK cells, as well as the involvement of nAChR β2, on the ability of these cells to perform immunosurveillance against murine B16 melanoma, a counterpart to a a highly prevalent and malignant cancer in humans.

Effective immune responses against lung tumor growth and metastasis rely on a strong activation of NK cells, CTLs as well as type 1 immunity. Weak antigenic stimuli by tumor antigens coupled with active suppression by regulatory cells preclude the difficulty in achieving effective anti-tumor immunity *in vivo*. Tumor intrinsic factors including reduced expression of class I molecule triggers NK cell cytotoxic activity. Tumor over growth, tissue damage and inflammation mobilize the cytokine synthesis machinery in NK cells. Early and burst release of IFN-γ in turn boosts type 1 immunity and CTLs and amplifies anti-tumor immunity. Suppression of both cytotocixic activity and cytokine production by NK cells essentially blunt two arms of NK cell –mediated anti-tumor immunity. Indeed, we found an significant increase in lung tumor burden in mice that received nicotine, and such an increase can be linked to compromised NK cell functions. In terms of melanoma, recognition of tumor cells by NK cells appears important to prevent metastasis. Expression of NK receptors NCR and DNAM-1 by both human and murine melanoma cells, as well as reduced expression of MHC-class I molecule, lead to recognition by NK cells and trigger the loss of NK cell tolerance. The importance of NCR is highlighted by evidence that NCR-expressing NK cell clones were required for the elimination of lung carcinoma and, conversely, loss of NRC expression on lung-resident NK cells led to tumor progression [15]. Whether nicotine directly affects expression of NCR or the accessory molecule DNAM-1 awaits further investigation. The lung metastasis model recapitulates the homogenous spread of melanoma. In adoptive transfer models, NK cells are sufficient to reduce tumor burden in the lung as well as other organs (data not shown).

Mammalian nAChR subunits are derived from a family of sixteen different genes (α1–α7, α9–α10, β1–β4, γ, δ and ε) and each have distinct distributions. The combinations and order of nAChR subunits within functional pentamers dictate nAChR subtype properties [25], [26]. Despite that nAChR expression has been examined in macrophages, dendritic cells, monocytes, T cells and B cells [27], expression prolife of nAChRs and their functions on NK cells have not been previously reported. Here we demonstrated that NK cells express nAChR α4, α5, α6, β2 and β3. In addition, positive ligand binding assay results imply nAChR β2 expression on NK cells may have biological functions. Indeed, influence of nicotine on NK cells, both at cellular and molecular levels, was largely abrogated in nAChR β2 deficient mice. The in vivo effects of nicotine in augmenting B16 metastasis become minimum in nAChR β2/RAG1 double knock-out mice, implying a crucial role for nAChR β2 in NK cells, which can not be compensated by other nAChRs. The role for α7 expression on macrophages in the peripheral immune system and microglia within the central nervous system in mediating the cholinergic anti-inflammatory effects are highlighted by recent studies [7], [8]. Despite the prominent role for nAChR α7, expression of other nAChRs appears to also influence the functions of macrophages and microglia [3], [4]. However, we found that only nAChR β2 appears as a prominent mediator for nicotine’s effects on NK cells. The overlapping expression pattern and their functional discrepancy of nAChR on macrophage and NK cells are interesting and deserve in depth investigation.

Taken together, our studies have revealed that NK cells express nAChR β2 which determine their function in controlling lung tumor metastasis in a murine model. These data reveal new insight in smoking-induced pathologies. Restoration of altered NK cell function induced by nicotine and smoking may possibly boost their capacity to prevent dissemination of early stage melanoma.

## Supporting Information

Figure S1 **Gross appearance of B16 tumor cell metastasis to lung in mice received PBS or nicotine.** Mice of different genotypes received nicotine (Nic) or PBS for 21 days and engrafted with B16 melanoma cells lines (1×10^6^ cells/mouse). Seven of 14 days later, a portion of mice were euthanized and the lung dissected. Representative pictures of lungs are shown.
(TIF)

Figure S2 **B16 melanoma cells dissemination in lung visualized by MRI T2 images.** Mice of different genotypes received nicotine (Nic) or PBS for 21 days and engrafted with B16 melanoma cells lines (1×10^6^ cells/mouse). T2 7MRI image denote B16 melanoma tumor cell dissemination in lung.
(TIF)

Table S1 **Comparison of numbers of lung metastasis in mice engrafted with different numbers of tumor cells.** Mice of different genotypes received nicotine (Nic) or PBS for 21 days and engrafted with B16 different numbers of melanoma cells lines as indicated. 14 of 21 days later, mice were euthanized and the lung dissected. Total numbers of melanoma nodules counted in these lungs are compared (n = 6 mice/group).
(DOC)

## References

[1] Sopori M (2002) Effects of cigarette smoke on the immune system. Nat Rev Immunol 2: 372–377. 12033743 10.1038/nri803

[2] Stampfli MR, Anderson GP (2009) How cigarette smoke skews immune responses to promote infection, lung disease and cancer. Nat Rev Immunol 9: 377–384. 19330016 10.1038/nri2530

[3] Wang H, Liao H, Ochani M, Justiniani M, Lin X, et al (2004) Cholinergic agonists inhibit HMGB1 release and improve survival in experimental sepsis. Nat Med 10: 1216–1221. 15502843 10.1038/nm1124

[4] Wang H, Yu M, Ochani M, Amella CA, Tanovic M, et al (2003) Nicotinic acetylcholine receptor alpha7 subunit is an essential regulator of inflammation. Nature 421: 384–388. 12508119 10.1038/nature01339

[5] de Jonge WJ, van der Zanden EP, The FO, Bijlsma MF, van Westerloo DJ, et al (2005) Stimulation of the vagus nerve attenuates macrophage activation by activating the Jak2-STAT3 signaling pathway. Nat Immunol 6: 844–851. 16025117 10.1038/ni1229

[6] van Maanen MA, Lebre MC, van der Poll T, LaRosa GJ, Elbaum D, et al (2009) Stimulation of nicotinic acetylcholine receptors attenuates collagen-induced arthritis in mice. Arthritis Rheum 60: 114–122. 19116908 10.1002/art.24177

[7] Shi FD, Piao WH, Kuo YP, Campagnolo DI, Vollmer TL, et al (2009) Nicotinic attenuation of central nervous system inflammation and autoimmunity. J Immunol 182: 1730–1739. 19155522 10.4049/jimmunol.182.3.1730

[8] Hao J, Simard AR, Turner GH, Wu J, Whiteaker P, et al. (2010) Attenuation of CNS inflammatory responses by nicotine involves alpha7 and non-alpha7 nicotinic receptors. Exp Neurol. 10.1016/j.expneurol.2010.09.020 PMC3019302 20932827

[9] Mian MF, Lauzon NM, Stampfli MR, Mossman KL, Ashkar AA (2008) Impairment of human NK cell cytotoxic activity and cytokine release by cigarette smoke. J Leukoc Biol 83: 774–784. 18055568 10.1189/jlb.0707481

[10] Tollerud DJ, Clark JW, Brown LM, Neuland CY, Mann DL, et al (1989) Association of cigarette smoking with decreased numbers of circulating natural killer cells. Am Rev Respir Dis 139: 194–198. 2912340 10.1164/ajrccm/139.1.194

[11] Lu LM, Zavitz CC, Chen B, Kianpour S, Wan Y, et al (2007) Cigarette smoke impairs NK cell-dependent tumor immune surveillance. J Immunol 178: 936–943. 17202355 10.4049/jimmunol.178.2.936

[12] Ng AK, Travis LB (2008) Subsequent malignant neoplasms in cancer survivors. Cancer J 14: 429–434. 19060610 10.1097/PPO.0b013e31818d8779

[13] Owens JC, Balogh SA, McClure-Begley TD, Butt CM, Labarca C, et al (2003) Alpha 4 beta 2* nicotinic acetylcholine receptors modulate the effects of ethanol and nicotine on the acoustic startle response. Alcohol Clin Exp Res 27: 1867–1875. 14691373 10.1097/01.ALC.0000102700.72447.0F

[14] Whiteaker P, Jimenez M, McIntosh JM, Collins AC, Marks MJ (2000) Identification of a novel nicotinic binding site in mouse brain using [(125)I]-epibatidine. Br J Pharmacol 131: 729–739. 11030722 10.1038/sj.bjp.0703616 PMC1572375

[15] Lakshmikanth T, Burke S, Ali TH, Kimpfler S, Ursini F, et al (2009) NCRs and DNAM-1 mediate NK cell recognition and lysis of human and mouse melanoma cell lines in vitro and in vivo. J Clin Invest 119: 1251–1263. 19349689 10.1172/JCI36022 PMC2673866

[16] Werneck MB, Lugo-Villarino G, Hwang ES, Cantor H, Glimcher LH (2008) T-bet plays a key role in NK-mediated control of melanoma metastatic disease. J Immunol 180: 8004–8010. 18523263 10.4049/jimmunol.180.12.8004 PMC3709580

[17] Robbins CS, Dawe DE, Goncharova SI, Pouladi MA, Drannik AG, et al (2004) Cigarette smoke decreases pulmonary dendritic cells and impacts antiviral immune responsiveness. Am J Respir Cell Mol Biol 30: 202–211. 12920055 10.1165/rcmb.2003-0259OC

[18] Hao J, Liu R, Piao W, Zhou Q, Vollmer TL, et al (2010) Central nervous system (CNS)-resident natural killer cells suppress Th17 responses and CNS autoimmune pathology. J Exp Med 207: 1907–1921. 20696699 10.1084/jem.20092749 PMC2931174

[19] Marks MJ, Smith KW, Collins AC (1998) Differential agonist inhibition identifies multiple epibatidine binding sites in mouse brain. J Pharmacol Exp Ther 285: 377–386. 9536034

[20] Whiteaker P, McIntosh JM, Luo S, Collins AC, Marks MJ (2000) 125I-alpha-conotoxin MII identifies a novel nicotinic acetylcholine receptor population in mouse brain. Mol Pharmacol 57: 913–925. 10779374

[21] McIntosh JM, Azam L, Staheli S, Dowell C, Lindstrom JM, et al (2004) Analogs of alpha-conotoxin MII are selective for alpha6-containing nicotinic acetylcholine receptors. Mol Pharmacol 65: 944–952. 15044624 10.1124/mol.65.4.944

[22] Lanier LL (2008) Up on the tightrope: natural killer cell activation and inhibition. Nat Immunol 9: 495–502. 18425106 10.1038/ni1581 PMC2669298

[23] Raulet DH, Vance RE (2006) Self-tolerance of natural killer cells. Nat Rev Immunol 6: 520–531. 16799471 10.1038/nri1863

[24] Smyth MJ, Taniguchi M, Street SE (2000) The anti-tumor activity of IL-12: mechanisms of innate immunity that are model and dose dependent. J Immunol 165: 2665–2670. 10946296 10.4049/jimmunol.165.5.2665

[25] Jensen AA, Frolund B, Liljefors T, Krogsgaard-Larsen P (2005) Neuronal nicotinic acetylcholine receptors: structural revelations, target identifications, and therapeutic inspirations. J Med Chem 48: 4705–4745. 16033252 10.1021/jm040219e

[26] Lukas RJ, Changeux JP, Le Novere N, Albuquerque EX, Balfour DJ, et al (1999) International Union of Pharmacology. XX. Current status of the nomenclature for nicotinic acetylcholine receptors and their subunits. Pharmacol Rev 51: 397–401. 10353988

[27] Piao WH, Campagnolo D, Dayao C, Lukas RJ, Wu J, et al (2009) Nicotine and inflammatory neurological disorders. Acta Pharmacol Sin 30: 715–722. 19448649 10.1038/aps.2009.67 PMC4002379

