# Supplementary figures and images for: Nicotinic Receptor β2 Determines NK Cell-Dependent Metastasis in a Murine Model of Metastatic Lung Cancer
Source: PLoS One. 2013 Feb 28;8(2):e57495. doi: 10.1371/journal.pone.0057495 (PMC3585320; doi:10.1371/journal.pone.0057495)

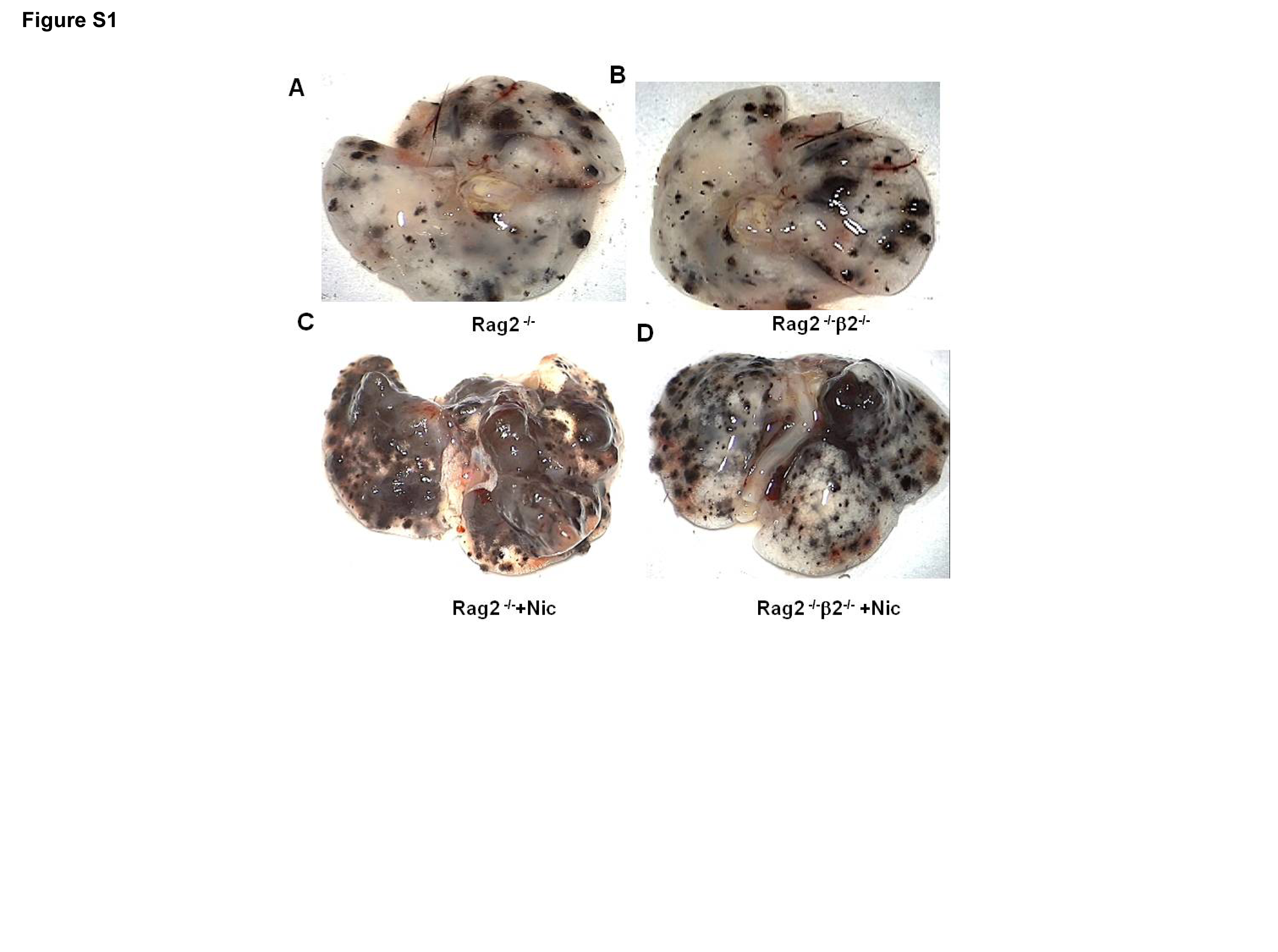

Supplement: Figure S1 — Gross appearance of B16 tumor cell metastasis to lung in mice received PBS or nicotine. Mice of different genotypes received nicotine (Nic) or PBS for 21 days and engrafted with B16 melanoma cells lines (1×10^6^ cells/mouse). Seven of 14 days later, a portion of mice were euthanized and the lung dissected. Representative pictures of lungs are shown. (TIF) [file pone.0057495.s001.tif]

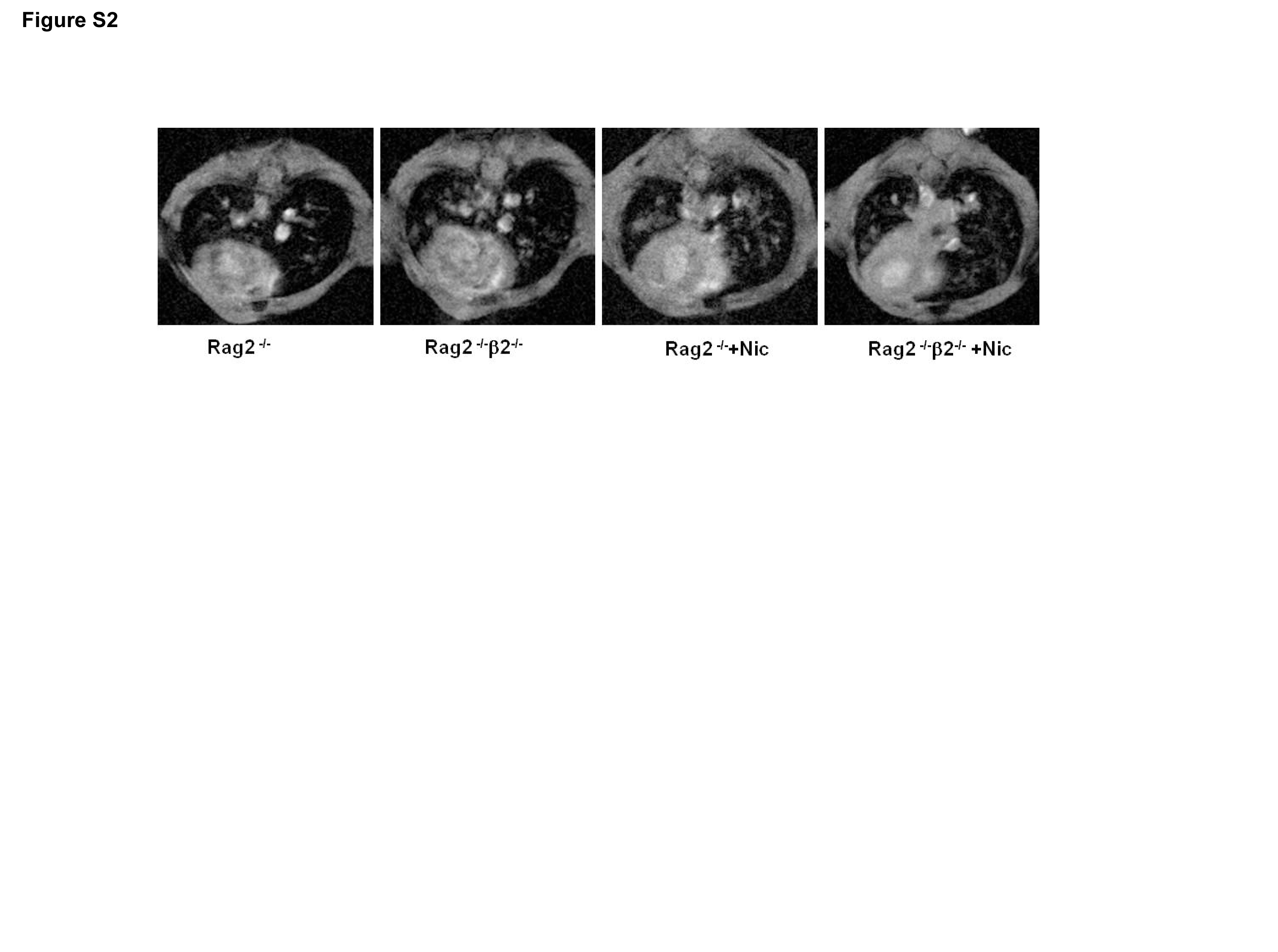

Supplement: Figure S2 — B16 melanoma cells dissemination in lung visualized by MRI T2 images. Mice of different genotypes received nicotine (Nic) or PBS for 21 days and engrafted with B16 melanoma cells lines (1×10^6^ cells/mouse). T2 7MRI image denote B16 melanoma tumor cell dissemination in lung. (TIF) [file pone.0057495.s002.tif]
