# Supplementary material for: Nicotinic Receptor β2 Determines NK Cell-Dependent Metastasis in a Murine Model of Metastatic Lung Cancer
Source: PLoS One. 2013 Feb 28;8(2):e57495. doi: 10.1371/journal.pone.0057495 (PMC3585320; doi:10.1371/journal.pone.0057495)
Supplement: Table S1 — Comparison of numbers of lung metastasis in mice engrafted with different numbers of tumor cells. Mice of different genotypes received nicotine (Nic) or PBS for 21 days and engrafted with B16 different numbers of melanoma cells lines as indicated. 14 of 21 days later, mice were euthanized and the lung dissected. Total numbers of melanoma nodules counted in these lungs are compared (n = 6 mice/group). (DOC) [file pone.0057495.s003.doc]

**Table S1. Number of lung metastases**

|  | | | **1x10^5^** | **2.5x10^5^** | **5x10^5^** | **1x10^6^** | **2.5x10^6^** |
| --- | --- | --- | --- | --- | --- | --- | --- |
| **Rag2^-/-^γc^-/-^** | **14d** | **PBS** | 70±15 | 100±19 | 139±33 | 178±20 | 182±31 |
|  |  | **Nic** | 75±23 | 106±21 | 145±26 | 180±29 | 189±35 |
|  | **21d** | **PBS** | 113±20 | 163±28 | 208±33 | 378±40 | 368±49 |
|  |  | **Nic** | 122±26 | 172±36 | 219±45 | 389±45 | 398±52 |
| **Rag2^-/-^** | **14d** | **PBS** | 39±9 | 48±12 | 60±10 | 102±13 | 108±26 |
|  |  | **Nic** | 45±11 | 46±16 | 65±13 | 110±15 | 103±30 |
|  | **21d** | **PBS** | 68±15 | 93±19 | 118±26 | 178±23 | 189±33 |
|  |  | **Nic** | 93±21 | 133±21 | 193±24 | 263±28 | 279±45 |
| **Rag2^-/-^β2^-/-^** | **14d** | **PBS** | 43±16 | 55±11 | 73±14 | 125±19 | 118±35 |
|  |  | **Nic** | 48±20 | 59±17 | 77±13 | 132±21 | 125±40 |
|  | **21d** | **PBS** | 62±18 | 88±16 | 120±19 | 183±25 | 172±37 |
|  |  | **Nic** | 89±31 | 116±23 | 164±24 | 275±29 | 264±39 |

**Table S1. Comparison of numbers of lung metastasis in mice engrafted with different numbers of tumor cells.**

Mice of different genotypes received nicotine (Nic) or PBS for 21 days and engrafted with B16 different numbers of melanoma cells lines as indicated. 14 of 21 days later, mice were euthanized and the lung dissected. Total numbers of melanoma nodules counted in these lungs are compared (n=6 mice/group).
